# Challenges with Simulating Modified RNA: Insights into Role and Reciprocity of Experimental and Computational Approaches

**DOI:** 10.3390/genes13030540

**Published:** 2022-03-18

**Authors:** Rebecca J. D’Esposito, Christopher A. Myers, Alan A. Chen, Sweta Vangaveti

**Affiliations:** 1 Department of Chemistry, University at Albany, State University of New York, 1400 Washington Avenue, Albany, NY 12222, USA; rjdesposito@albany.edu (R.J.D.); achen6@albany.edu (A.A.C.); 2 Department of Physics, University at Albany, State University of New York, 1400 Washington Avenue, Albany, NY 12222, USA; cmyers2@albany.edu; 3 The RNA Institute, University at Albany, State University of New York, 1400 Washington Avenue, Albany, NY 12222, USA

**Keywords:** RNA, RNA modifications, molecular dynamics, parameterization

## Abstract

RNA is critical to a broad spectrum of biological and viral processes. This functional diversity is a result of their dynamic nature; the variety of three-dimensional structures that they can fold into; and a host of post-transcriptional chemical modifications. While there are many experimental techniques to study the structural dynamics of biomolecules, molecular dynamics simulations (MDS) play a significant role in complementing experimental data and providing mechanistic insights. The accuracy of the results obtained from MDS is determined by the underlying physical models i.e., the force-fields, that steer the simulations. Though RNA force-fields have received a lot of attention in the last decade, they still lag compared to their protein counterparts. The chemical diversity imparted by the RNA modifications adds another layer of complexity to an already challenging problem. Insight into the effect of RNA modifications upon RNA folding and dynamics is lacking due to the insufficiency or absence of relevant experimental data. This review provides an overview of the state of MDS of modified RNA, focusing on the challenges in parameterization of RNA modifications as well as insights into relevant reference experiments necessary for their calibration.

## 1. Introduction

The canonical four-letter code that comprises RNA is no longer sufficient to capture the abundance of information that RNA can convey. Over 140 naturally occurring modifications of adenosine (A), guanosine (G), cytosine (C), and uracil (U) have been discovered to date [1] and have been found in all types of RNA including transfer RNA (tRNA) [2], messenger RNA(mRNA) [3,4], ribosomal RNA (rRNA) [5,6], as well as all life forms (archaea [7], bacteria [7,8], and eukarya [9]) and even in viruses [10]. Furthermore, synthetic nucleic acid analogs have been explored and utilized by scientists in developing antiviral drugs [11], nucleic acid-driven therapeutics [12], and mRNA vaccines [13].

The first modified nucleotide, pseudouridine, was discovered in *Saccharomyces cerevisiae* in the 1950s [14], but barring sporadic discoveries, five decades passed before interest revived and developments in modified RNA research renewed. Studying RNA modifications has many obstacles, of which the most challenging is detection. RNA modifications are present in cells at extremely low quantities and can have high turnover, where they are frequently erased or further altered by proteins within the cell [15]. Therefore, collecting relevant data that can characterize RNA modifications typically requires multiple techniques, both experimental and computational, to work in concert. The emergence of genomic techniques has made large amounts of transcriptome-wide data accessible, which has, in turn, accelerated the study of RNA modifications in the past two decades [16,17,18]. However, the analytical tools necessary for the detection, identification, and quantification of RNA modifications are still in their infancy. 

Not surprisingly, researchers are still scratching the surface when it comes to discerning the biological relevance of RNA modifications. Several modifications have now been linked to disease pathologies [9], stress pathways [19,20], neuro-regulation [21], gene expression and regulation [22], and fetal development [23], amongst others. However, a mechanistic understanding of how modified nucleotides affect cellular processes and pathways has yet to be attained. Experimental techniques like X-ray crystallography, nuclear magnetic resonance spectroscopy (NMR) or cryogenic electron microscopy (Cryo-EM), and computational techniques like atomistic molecular dynamics simulations (MDS) are invaluable in providing insights into such mechanisms. In this review, we discuss the successes and shortcomings of these techniques vis-à-vis probing structure–function relationships of modified RNA. 

## 2. Classification of Modified RNA Nucleosides Based on Their Structural/Functional Implications

**Figure 1:**
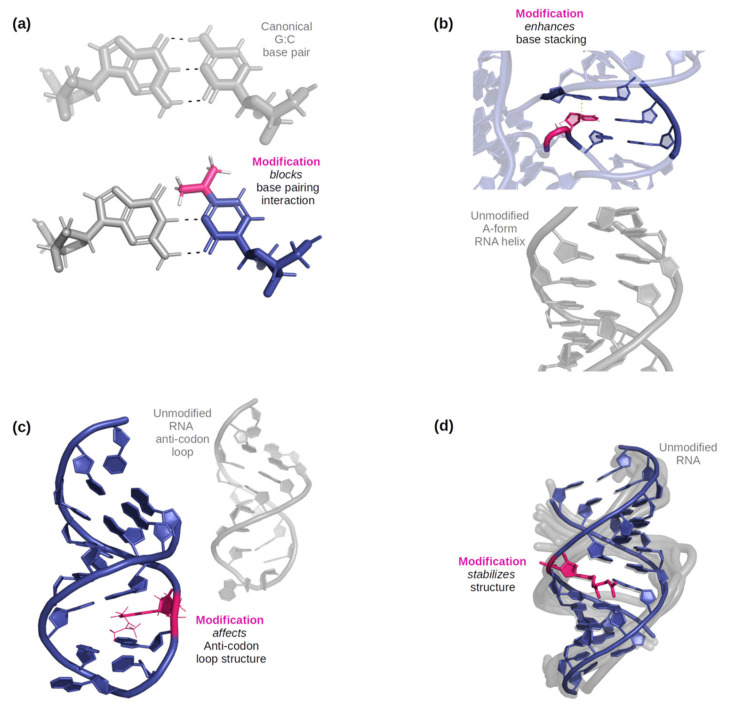
(**a**) An example of how a modification can affect base-pairing interactions. Here, *N^4^*, *N^4^*-dimethylcytidine (in pink) has only two possible base-pairing sites on its W-C-F edge due to the double substitution of methyl groups on the amine, while a typical G: C base pair would have three [36]. (**b**) An example of three base pairs, one only with canonical bases, and the other with one RNA modification (5-methylcytidine, illustrated in pink). The dashed line indicates where the methyl group would help stabilize stacking with the nucleobase above it [37]. (**c**) An example of a modified anticodon loop structure vs an unmodified anticodon loop. Due to *N^6^*-isopentenyladenosine (in pink), an additional base pairing occurs below the modification and the nucleotides in the loop become more stable as it became smaller [38]. (**d**) An example of an effect on helical stability due to the presence of 2-geranylthiouridine (shown in pink) [39].

Before delving into the methods used to study modified RNA structure and dynamics, it is important to appreciate the chemical and structural diversity of the RNA modifications. From a structural perspective, the two main factors that drive RNA folding pathways are base stacking and base pairing, which are achieved by hydrogen bonding (h-bond) interactions [24,25]. Base pairing can occur in different orientations based upon which ‘edge’ of the nucleobase is involved in the pairing [26]. Base stacking contributes to duplex formation as well as the stability of a folded RNA [27,28]. RNA modifications contribute to either enhanced, reduced, or altered base pairing and stacking preferences, conformational flexibility, helical winding, groove hydrophobicity and polarity, and stability of tertiary and long-range interactions [1,22,29]. A few examples of these phenomena are illustrated in Figure 1. Acknowledging these factors, we have classified the naturally occurring RNA modifications based on how their position within the nucleotide and their chemical properties can alter the structural and consequently biological behavior of RNA. 

### 2.1. Based on the Location of the Modified Group in the Modified Nucleotide

**Figure 2:**
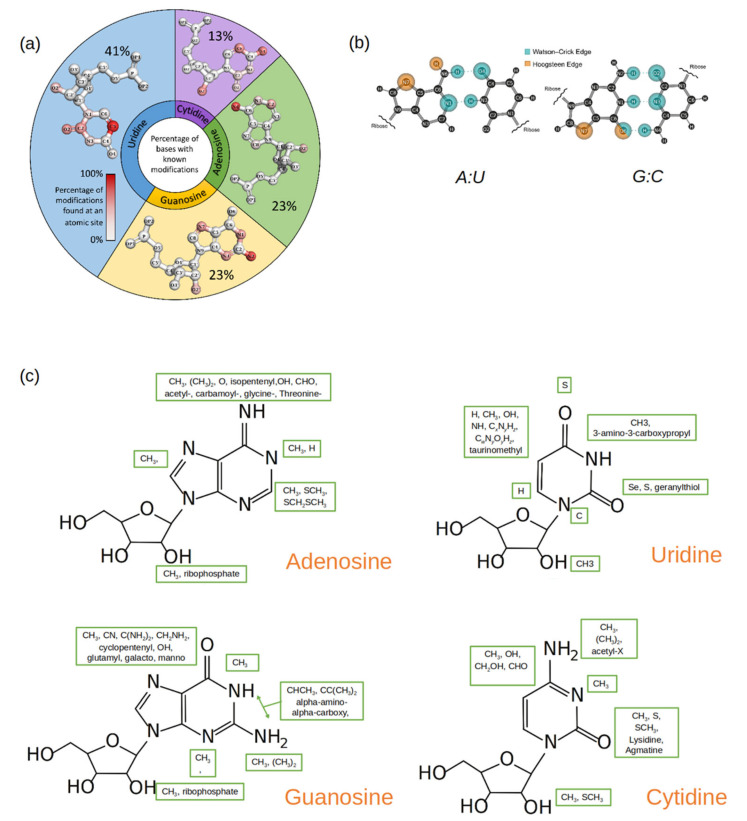
(**a**) A pie plot where each section represents a canonical nucleotide (A, C, G, U) and the size of each section reflects the percentage of the naturally occurring RNA modifications that originate from that canonical nucleotide. Within each pie section, the structure of the canonical nucleotide is displayed, and the atom positions are colored by gradient, which is based upon how frequently that position is modified. (**b**) Standard A: U and G: C base pairs with the Watson–Crick (blue) and the Hoogstein (orange) base pairing edges highlighted. (**c**) Common functional groups (enclosed in green boxes) that occur at different atomic sites in modified nucleotides. The structure of the parent nucleotide is used as a reference.

Out of the 143 naturally occurring RNA modifications that are currently listed in the Modomics database [30], 57% contain modifications that affect the Watson–Crick–Franklin (W-C-F) edge, 46% contain modifications that affect the Hoogstein/“C-H” edge, 0.7% contain modifications that affect the backbone (phosphate), and 20% contain modifications that affect the sugar edge. Many RNA modifications have multiple sites modified and are represented as such in the percentages. In Figure 2a, the sites of possible modification are shown on each of the four canonical RNA nucleotides using a gradient scale, red indicating a site of frequent modification while gray indicates a site of less frequent modification. For G, A, and C, the site of most frequent modification falls on the W-C-F edge, while on U, the site that is most frequently modified falls on the “C-H” edge.

Among the purines (A and G), the hydrogens in the amine group are most frequently substituted by one or two methyl groups. Since the amine group is on the W-C-F edge (Figure 2b), methylations at this site (position 6 on A and position 2 on G) affect the purines’ base-pairing preferences [31,32,33,34,35]. Methylations at other sites on the purines’ W-C-F edge will also affect potential base pairings as well as influence base stacking [22]. 

Modifications on C comprise only 13% of the total RNA modifications discovered thus far. In the current data set, there are more cases where positions 4 and 5 on the pyrimidine ring in C are modified, followed by positions 2 and 3. While the modified groups at positions 2, 3, and 4 change the base-pairing face of C, the 5th position modifications have shown to improve duplex stability [40].

U is the most commonly modified nucleotide in RNA, and many of these modifications are found at position 5 of the pyrimidine ring. Unmodified, U forms a canonical and a wobble base pair with A and G respectively. Modifications that occur at position 5 can shift the base-pairing preference [41,42]. Furthermore, A: U and G: U pairs are demonstrably weaker than the canonical G: C pair, and modifications at position 5 in U can be used to add stability to the base pairs without changing the base-pairing edge [43,44]. Position 2 on U is the second most common site to be modified. Unlike the modifications at position 5, this modification can directly influence the base-pairing preference of U, specifically because it is involved in base-pairing with A but not G [45,46].

The ribose is a moiety found in every nucleotide, modified or unmodified. An unmodified ribose can have two configurations: C2′-endo sugar puckering or C3′-endo sugar puckering [47]. Typically, RNA nucleotides have their ribose in the C3′ endo sugar pucker as this allows for RNA to assemble A-form helices and single-stranded regions. DNA nucleotides, however, have their ribose moieties in the C2′-endo sugar pucker form, as the hydroxyl group in the 2′ position of the ribose in RNA is replaced by hydrogen in DNA. This slight difference has an impact on the sugar structure and, not surprisingly, many sugar modifications in RNA occur at the 2′ position on the ribose [48]. 2′O methylations are especially common and have been found in all major RNA groups [1,22,47]. Methylation at the 2′ position can cause changes in hydrogen bonding characteristics and can weaken the glycosidic bond between the ribose and the nucleobase [47]. It can also disrupt interactions of RNA that depend on the hydroxyl group at the 2′ position [49]. Another type of ribose modification is the addition of a second ribose group between the phosphate and the backbone. These ribosyl RNA modifications (Ar(p) and Gr(p)) were first found in tRNA but have now been linked to proteins and metabolites [50]. As there is a second ribose with its sugar pucker form, this bulky modification can significantly affect stacking specifically through distorting the phosphodiester backbone [50]. 

### 2.2. Based on the Nature of the Modified Group in the Modified Nucleotide

The ever-expanding universe of known RNA modifications also displays remarkable chemical diversity (Figure 2c). The modifications range from simple methyl groups to elaborate groups containing glycosylations, carboxylations, long straight or branched carbon chains (geranylation), and ring rearrangements (pseudouridine) [1,48]. This chemical diversity is achieved through site-specific enzymatic addition and removal by writer, reader, and eraser proteins using a myriad of reactions, such as methylations, thiolations, glycosylation, isomerizations, and deaminations [1,48].

It is no surprise that methylation is the most common modification found in all four nucleotides at various positions including the sugar, considering the framework to introduce methylations is well established and methylations are common epigenetic markers and post-translational modifications [1,22,48]. In fact, of the naturally occurring RNA modifications, 68% of modified adenosines, 55% of modified guanosines, 50% of modified uridines, and 38% of modified cytosines contain at least one methylation. Structurally, a simple methylation affects a possible hydrogen bond donor or acceptor site. In the case of methylations along a base-pairing edge, the methyl group essentially blocks the pairing from occurring [22,51,52]. Depending on the sequence position of the methylated nucleotide, this modification can enhance base stacking as well [22].

After methylations, groups such as hydroxyls (OH), thiols (S), and amines (NH_2_) are the next most common type of RNA modification, depending on the original canonical nucleotide. These modification groups can add hydrogen donors and acceptors to the base pairing edges [1]. This can lead to wobble pairing, a non-typical base-pair conformation, or a different preferred pairing partner (e.g., G: U) [1,22,51,53]. Specifically, thiolated uridines (such as s^2^U and s^4^U) have been shown to base pair with a wide range of partners as well as affect thermostability by enhanced stacking interactions [51].

In addition to modification of nucleobase ring substituents by small chemical groups, there exist RNA modifications where the purine and pyrimidine ring structure is altered. Pseudouridine is the most commonly found RNA modification and is significant as pseudouridine is one of the few, if not the only ring rearrangement-based naturally occurring RNA modification. It is an isomer of uridine in which the base is attached to the sugar via a C-glycosidic bond, instead of an N-glycosidic bond. The C-C bond gives the nucleobase more rotational freedom and conformational flexibility [1,22,52]. The 180° ring rotation also allows for an extra hydrogen bond donor at the N1 position. This ring rearrangement allows pseudouridine to sample different pairing and possibly stabilize the structure of the RNA when utilizing the extra hydrogen bond donor at the N1 position [22,52,54]. In wyosine, wybutosine, and their derivatives, the purine ring of G is extended to include a third ring. Little is known about the structural properties of the tricyclic ring or the accompanying large side chains of these modifications that assist in maintaining the reading frame during translation [55]. Some molecular modeling studies suggest that restricted conformation sampling induced by the modification and its bulky side chains could be responsible for stabilizing codon–anticodon interactions and indirectly affecting translation [56,57]. 

Some more exotic RNA modifications include glycosylated, geranlyated, and amino acid-based RNA modifications. Glycosylated RNA, or glycoRNAs, are a recent discovery and have been found in multiple cell types and mammalian species [58]. GlycoRNAs are modified with complex-type N-glycans with at least one terminal sialic acid residue and have been determined to interact with surface proteins and antibodies [58]. Geranylated RNA nucleotides (e.g., 2-geranylthiouridine) are very hydrophobic and have been found to disrupt the helical structure and affect base pairing [39]. Amino acid-based RNA modifications make up a unique group compared to the rest of the naturally occurring modified RNA nucleotides. Amino acid-based modifications are unable to establish base pairing, however, they can incorporate and interact with other amino acids and proteins [59]. Structurally, they are bulky and can only ‘fit’ into structural motifs that have enough ‘room’ (e.g., loops, bulges, junctions) [59]. 

### 2.3. Summary of the Classification of RNA Modifications

The location of the modification on a nucleotide and the actual chemical group together determine how a chemical modification influences the structural behavior of the modified RNA. The location-based categorization of the modifications yields two main categories—nucleobase and backbone modifications. The nucleobase modifications can be further split into W-C-F and CH edge modifications, while the backbone modifications can either occur on the ribose or the phosphate groups. Based on the chemical nature of the modification, the modifications can be classified into simple and complex substituents, reorganized ring structure, and modifications shared with other biomolecules. In addition to the obvious shift in base-pairing and base-stacking propensities of the nucleotides due to nucleobase modifications, all modifications have the potential to affect the stability and conformational flexibility of the RNA.

So far, there has been minimal insight into the molecular details of how modifications affect the structural and functional aspects of RNA, from both experimental and computational efforts. However, one can conclude that RNA modifications do have the potential to significantly affect the structure, and as a result, the biological functions of RNAs. Perhaps, by leveraging the strengths and weaknesses of both computational and experimental efforts, these mechanistic effects can be gleaned.

## 3. Molecular Dynamics Simulations (MDS) of Modified RNA

MDS are an extremely useful computational tool to elucidate how the “wiggling and giggling” of atoms gives rise to the folding pathways, three-dimensional (3D) structure, and interactions of biomolecules. The two key components needed for reliable MDS are accurate initial 3D coordinates and robust “force-field” parameters, which steer the simulation over time to explore energetically favorable conformations. Force-fields are a collection of analytical functions and their associated model parameters that estimate the intra and intermolecular forces between atoms and molecules in MDS. It is standard practice for MDS to use experimentally solved 3D structures from databases like the Protein Data Bank (PDB) [60] to study how equilibrium fluctuations of the pre-folded biomolecules explain its biochemical function. For simulations of RNA that involve the study of its interactions with proteins or small molecules, in the absence of an experimentally determined structure of the complex, molecular docking can be used to generate initial guess structures. This eliminates the need for the exhaustive simulations required to fold an RNA sequence *ab-initio* or for the interacting molecules to find a suitable orientation, an endeavor which would not be expected to succeed at this point in time due both to imperfections in available force-fields and the lack of sufficient computing power to propagate simulations to relevant millisecond (ms) to second (s) timescales. 

However, obtaining a reasonable initial 3D structure is often a bottleneck, as only 7% of total structures deposited in the PDB contain RNA and only a small fraction of those contain any RNA modifications (naturally occurring or synthetically derived). While strategic modeling and advanced sampling techniques can somewhat alleviate the shortage of acceptable initial RNA structures, they cannot overcome the inability of current force-field parameters to depict inter and intramolecular interactions of RNA accurately. This obstacle is enough to prevent the achievable folding of RNA sequences into their characteristic 3D structures in silico. Fortunately, there is rapid ongoing progress in the development of improved force-field parameters for unmodified RNA, which have been recently updated with RNA-specific improvements in several popular force fields [61,62,63]. However, current iterations have only proven successful in capturing folding and dynamics of small unmodified RNA tetramers and tetraloop hairpins. MDS studies undertaken to understand the behavior of even medium-sized RNAs (>10 *nucleotides*) encounter several challenges as has been pointed out in recent reviews [64,65]. Due to the limitations with simulating unmodified RNA and the sheer number of known RNA modifications, there have only been a handful of attempts to develop force-field parameters for modified RNA nucleotides. Xu et al. [66] and Aduri et al. [67], for example, have published works containing parameters for over 100 different RNA modifications each. Parameters for some modifications can also be obtained from websites like the Bryce Lab’s AMBER parameter database [68] or published work on simulations of modified RNA [39,69]. These sources primarily extend two popular force-field parameterization strategies (AMBER and CHARMM) to include modified RNA nucleotides in a manner that is self-consistent with how the canonical RNA nucleotides were parameterized. However, this does not guarantee that no further calibrations are needed, as discussed in the brief overview of parameterization strategies below.

### 3.1. Force-Field Parameterization Strategies 

**Figure 3:**
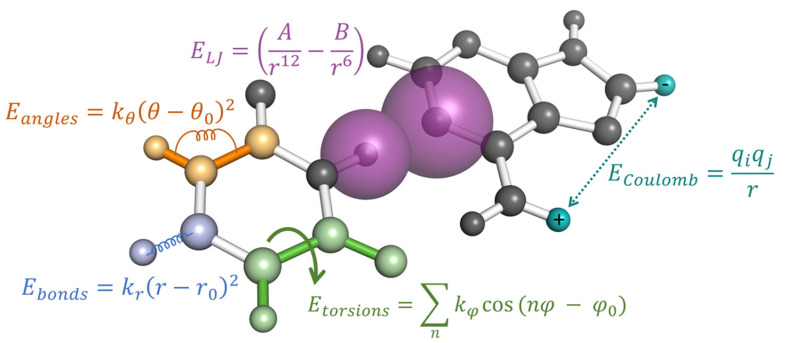
The potential energy of an MD simulation is calculated using pairwise additive energies as a function of their geometric distances and angles relative to other atoms. Each type of interaction is represented by a single example in this figure, while the total energy of the system is the sum over all bonded terms ($E_{bonds},\;E_{angles}$, and $E_{torsions}$) and non-bonded terms ($E_{LJ}$ and $E_{Coulomb}$).

MDS incorporate two major types of molecular interactions as depicted in Figure 3 bonded terms that dictate the stretching and bending of covalent bonds at short atomic distances and nonbonded terms that describe both the inter and intramolecular non-covalent interactions at the intermediate to long ranges. Regardless of the force-field chosen, the bonded terms are typically calculated in a “ball and spring” manner, where each atomic nuclei is attached to another via a harmonic Hookean spring to replicate the energy associated with covalent bond stretching and compaction. This is further extended to include the energetic contributions associated with the relative movement of second and third nearest neighboring atoms through bond angle and torsion rotations, respectively. Equilibrium bond distances can be obtained from high-resolution crystal structures when available, while the spring constants dictating the stretching magnitude are typically taken from ab initio harmonic frequency calculations [70]. As many of the bonded interactions can be easily verified against experimental spectroscopy data [71,72], these parameters are considered to be accurately transferable between most biomolecular force fields. They can also be extended to modified RNA since most of the known RNA modifications are composed of common chemical moieties that have already been parameterized.

The fitting of nonbonded interactions, on the other hand, are much more varied between different force fields. The philosophy behind each parameterization strategy will have a greater impact on how the modified RNA nucleotide will behave in MDS. Classical, all-atom simulations of biomolecules typically include two separate nonbonded forces: Coulombic interactions between charged atomic sites and Lennard-Jones forces to account for electron overlap and correlational effects. Coulombic interactions are described by static, atom-centered point charges, while Lennard-Jones forces are described by a pairwise additive inverse power law consisting of attractive 6th power and repulsive 12th power components (see Figure 3). These nonbonded forces are intended to reproduce the inherently quantum mechanical (QM) phenomena of interacting atoms and molecules, such as short-range Pauli exchange repulsion, mid-range London dispersion between correlationally polarized electrons, and long-range frozen electrostatics. Collectively, these terms dictate the steric collision distances between all parts of the RNA as well as weak attractions, such as base stacking, and are mostly determined by their hybridization state or from isolated nucleotide fragment geometries. 

It should be emphasized that all these functional forms were historically chosen purely based on computational convenience and less because of any deep connection to the underlying quantum-mechanical phenomena they are intended to represent. Electrons are not well-described as static point charges embedded in the center of atoms (even to a first approximation), and dispersion forces are inherently multi-body, environment-specific interactions that are not well described by static, spherically symmetric pairwise additive functions. Together, however, these functional forms provide an ample parameter-space for creating simple atom-centered classical models that can reproduce a wide range of physical phenomena with a small number of carefully calibrated parameters (notably, the extent to which the complex behaviors of water can be faithfully modeled by simple three or four-point models should be considered a marvel of computational chemistry [73,74,75,76,77]. However, the inherent coupling of Lennard-Jones with Coulombic energies also leads to ambiguity on how strong interactions such as hydrogen bonding or salt bridges should be balanced by each energy function. Knowledge of the ground-state geometry of a compound provides insufficient information on how to calibrate the effective strengths of these two terms most accurately unless additional external constraints are imposed. Consequently, for modified nucleotides, these parameters are often directly transferred from standard nucleotide parameters, and only the atomic charges are re-fitted to describe the remainder of the nonbonded interactions [78,79], which again is an assumption borne largely out of convenience. It is here where the fitting strategies for RNA force fields diverge significantly. Two of the most popular parameterization philosophies, AMBER and CHARMM, use different methods for fitting the atomic charges, and it is worth commenting on the differences and possible limitations between the two fitting strategies. 

### 3.2. Modified Nucleotide Parameterization Strategies for AMBER and CHARMM

Among the AMBER-based parameterizations, Aduri et al. [67] published parameters for 107 naturally occurring modified RNA nucleotides, including both sugar and nucleobase modifications. In the spirit of the AMBER pipeline, their work took on a modular approach, focusing mostly on deriving atomic partial charges and transferring the remaining parameters from GAFF (Generalized Amber force-field) [80]. The AMBER approach for deriving atomic charges is to replicate the electrostatic potential (ESP) produced by a molecule’s nuclei and quantum mechanical electrons with atom-centered point charges [81]. This is done by calculating the ESP at various positions around the molecule of interest using Hartree–Fock-based calculations (specifically HF/6-31G*) and adjusting the atomic charges until the ESP is replicated by the force field. The philosophy for this calculation is rather straightforward: If two point charges interact via their electrostatic potentials and if the charges can accurately reproduce a QM-derived potential, then the two atomic sites should, in principle, share the correct QM interaction energy. Although this may be more true at large distances from the atomic centers, there is no guarantee that this is true for all points in space, especially at close ranges when electron overlap can occur. Additionally, the instantaneous polarization from each atomic site is also not incorporated into these calculations. Nevertheless, the choice of combined Hartree–Fock exchange with a 6-31G* basis set is known to artificiality “pre-polarize” the charges [78] and is explicitly chosen to approximate these effects in a premeditated fashion. While other QM methods based on density functional theory (PBE, B3LYP, etc.) or perturbation theory (MP2) may produce similar charges, the same QM method used by the original AMBER parameterization is used by Aduri et al. [67] to maintain compatibility with the remaining charges in the canonical version of the force-field.

The CHARMM approach to charge fitting, and to the fitting of the majority of their force-field parameters, is to globally optimize all non-bonded parameters together until specific ab initio quantities or experimental data are accurately reproduced by the force-field [82,83]. As performed by Xu et al. [66], common choices of these include QM energies obtained from MP2 geometry optimized structures, the non-bonded interaction distances of these geometries and their electric dipole moments, and experimental crystal structures. For the QM quantities, potential energy profiles are performed with a single water molecule interacting via the possible hydrogen bonding sites of both the modified base and sugar. As MDS are driven by the derivatives of their molecular mechanics energy profiles, the benefit of the approach is that the resulting force-field is explicitly parameterized with ab initio profiles in mind, whereas the AMBER approach only implicitly attempts to get these quantities correct via the ESP fit, albeit their dependence on fundamental electrostatics. Like AMBER, CHARMM also attempts to implicitly account for polarization effects from water through phenomenological scaling factors applied to the ab initio energies and dipole moments produced by HF/6-31G* calculations [84], and Xu et al. continued with this tradition for their modification parameters.

Many of the versions of CHARMM have historically involved an iterative and highly structured fitting procedure in which all parts of the force-field, including bond distances, charges, torsion angles, and sometimes Lennard-Jones parameters, are continuously adjusted until the included QM and experimental data are reasonably reproduced by the MM force-field [85]. For this reason, the CHARMM community has honored a stricter definition of what is considered an addition to their force fields. Luckily, many prospective users may not need to forgo this parameterization process, as parameters for modified nucleotides are publicly available via the MacKerell Lab’s website [86], while the remaining standard atom types can be taken from the CGenFF (CHARMM General Force Field) program [79].

### 3.3. Molecular Dynamics Simulation Studies of Modified RNA

Molecular dynamics simulations can prove extremely useful in providing atomistic details on interactions, structural mechanisms, and the biological implications of RNA modifications. However, MDS of modified RNA has been limited by the imperfections of the force fields of RNA and the lack of experimental data needed for their calibration. At this time, it is nearly impossible to capture or quantify accurately the effects of modifications on large structural rearrangements of RNA via MDS. However, qualitative comparison with validating experimental evidence can be used to understand the effects of modifications on their localized inter and intramolecular interactions, and conformational stability in defined structural contexts. Some examples of defined structural contexts include modifications in the tRNA, in the codon–anticodon mini-helix, in standard A-form RNA duplexes, etc. Among the few structures of naturally occurring modified RNA in the PDB, a majority belong to independent, synthetase-bound, or ribosome-bound tRNAs. 

The fully modified X-ray crystal structure of tRNA^Phe^ [37] and the readily available AMBER parameters for its modified nucleotides from the database maintained by the Bryce Lab [68] have served as a robust system and act as a foundation for studying how modifications affect overall RNA dynamics. Specifically, studies on tRNA dynamics ([87] and how individual modifications alter the conformational landscape of the nucleotides to induce localized structural changes [57,69,88]) have benefited from this model system. 

Another common modified RNA system that is explored using MDS is the tRNA: mRNA minihelix in the context of the ribosome and the effects of modified nucleotides on codon-bias, and frame-shifting. Experimental observations provide evidence of the significance of the modification status of anticodon stem-loop (ASL) of tRNAs during translation and MDS studies alongside available experimentally derived structures, furnishing mechanistic insights for such observations. For example, codons NNA or NNG can be identified by the same tRNA with anticodon UNN, where N can be A, C, G, or U. Such systems have been shown to rely on modifications at the 34th and 37th positions in the tRNA to introduce codon bias. In eukaryotes, mcm^5^U_34_ and ms^2^t^6^A_37_ are both necessary for the ASL of tRNA^Lys^_UUU_ to successfully recognize the AAG codon. MDS showed that the methyl-thio group enhances the stability of the codon:anticodon minihelix by additional stacking interactions while the threonyl group shields the codon and the anticodon from the solvent, thus stabilizing the wobble G:U base pair in the AAG bound conformation of the tRNA [89,90]. In contrast, in the case of tRNA^ILe^, where Lysidine and t^6^A are at the 34th and 37th positions respectively, MDS show that the Lysidine preferentially pairs with AUA instead of AUG [91]. In bacteria, tRNAs with geranylated-2-thio uridine (ges^2^U) in the 34th position were shown to recognize only G-ending codons. MDS of the mRNA bound tRNA^Lys^ ASL showed that the loss of a proton donor due to geranylation in uridine prevents the A:ges^2^U pair from forming. However, the G:ges*^2^*U pair interacts with stable hydrogen bonds in the presence of the modification, and the bulky geranyl group does not disrupt any ribosomal interactions of the tRNA or mRNA [39]. A 2′O methylation in the coding region of mRNA has been shown to inhibit translation, which, one MDS study suggests could be a result of disrupted interactions between the mRNA and ribosomal RNA at the ribosomal A-site [49]. 

It is important to point out that although the MDS studies listed above are in qualitative agreement with experimental observations, they are somewhat speculative when providing mechanistic insights into the effects of the modifications on RNA structure and function. The force-field parameters used in these studies are acquired using the AMBER or CHARMM methodology and are only as good as those for the canonical RNAs. 

### 3.4. Summary of MDS of Modified RNA

There is no intrinsic reason why either the AMBER or CHARMM methodology should be better suited for simulating modified RNAs. Although the two parameterization strategies are quite distinct, there is no unique mapping of the inherently many-body quantum interaction energies into a classical, pairwise additive effective potential. Both strategies have evolved to incorporate calibrations or assessments against experimental data when available but must resort to fitting against gas-phase QM interaction energies in the absence of such data. Both Xu et al. [66] and Aduri et. al. [67] deliberately noted the limitations in their models arising from the much smaller amount of experimental information available for modified nucleotides as compared to their canonical counterparts. While both works aim to capture important topological properties of modified RNAs, such as backbone torsional populations or the replication of small crystal structures containing modified nucleotides, it is yet to be seen how each of these models perform when compared against additional thermodynamic or energetic experimental information. Still, that is not to say that ab initio-based calculations are not useful tools for developing modified force fields, but the question always remains how transferable gas-phase QM calculations on minimal molecular fragments can accurately reflect the behavior of macromolecules immersed in a physiological milieu of water, ions, and other biomolecules.

The degree to which the parameterization strategy may matter scales directly with the chemical nature of the modification. If the modification mostly involves space-filling or nonpolar additions such as methylations or other simple hydrocarbon groups (many of which are shown in Figure 2c), then the additional charge due to the modified group will essentially be zero and relatively insensitive to the method of charge assignment. The lack of polar groups means that polarizations are less likely to dominate intermolecular interactions, and direct comparison against ab initio-derived geometries may provide enough information for the adjustment of Lennard-Jones radii or potential strengths [62]. Even when strong electron correlation effects are at play, such as dispersion-mediated aromatic stacking, many modern DFT functionals can accurately predict equilibrium energy locations at a significantly less cost than MP2 or other highly correlated wave-function based calculations [92,93], resulting in the possibility of direct calibration of force-field parameters against gas phase QM interaction energies without needing any experimental data in the parameterization process.

However, if significantly polar modifications are involved, particularly in ones that alter the number or strength of hydrogen bonding sites, then the quality of the fit will depend more upon the exact atomic charges. Consequently, a more strategic approach based on each force-fields’ original parameterization philosophy is needed to balance the strength of intermolecular forces between charge-charge and van der Waals dispersive interactions. It should be noted there is no best method, a priori, to decompose intermolecular interaction energies into classical “spherical cow” terms such as point charges and van der Waals forces, even if neglecting higher-order terms such as polarization, which are typically ignored in classical force-fields. In such scenarios, it is essential to have experimental data suitable for direct parameter calibration, as interaction strengths between polar moieties are very environment specific. For biomolecular force fields, any highly polar group will interact strongly with aqueous solvent as well as with ions, greatly reducing the applicability and suitability of parameterizing solely against gas-phase QM calculations. When experimental data does not exist (for example, solvation free energies, conformational preferences, base-pairing thermodynamics), the simulator must rely on their chemical intuition for how strong or weak bonding should occur for each modification relative to their canonical interactions. In such a case, ab initio interaction energies can still be a useful gauge, however, unless some sort of scheme is used to account for how the fragments interact in a solvated environment via a thermodynamic free energy calculation, these calculations should only be considered as a qualitative and not a quantitative measure of accuracy given the potential complexity of the interaction.

Lastly, some modification groups may introduce a net charge or additional rotatable bonds, and these necessitate additional considerations. Net charged moieties would be expected to interact strongly with counter-ions, depending on how solvent-exposed and localized the charge is distributed. RNA itself is famously a polyelectrolyte whose behavior is altered significantly depending on both the identity and the concentration of the counter-ions present (especially divalent ions); therefore, modifications that affect the overall charge of the nucleic acid may also exhibit ion-dependent behavior requiring additional calibration. In terms of rotatable bonds, simple modifications resembling the set of organic compounds used in the genesis of early force fields [94,95] likely will perform adequately with generic model compound torsions recycled from existing parameters without needing further refinement. However, rotatable bonds in more exotic moieties may require custom torsional potentials calibrated against either QM/DFT interaction profiles or experiments that reveal conformational preferences such as NMR to ensure that the different rotamers are accurately sampled in the simulation. 

## 4. Experiments That Can Help Validate MD Simulation Results 

Many different analytical techniques are available to study modified RNAs. However, not all techniques afford data that is directly comparable to simulation results for parameter calibration or overall assessment of simulation accuracy. Below, several experimental techniques that are commonly used to investigate modified RNA systems are discussed. First, detection methods are addressed, as identifying a possibly modified position in the sequence of a RNA is critical as well as chemically identifying the modified nucleotide. Then, the focus turns to structure analysis methods, as ultimately, evaluating the structural effects of RNA modifications on a biological RNA will answer inquiries into its function. Each technique is outlined, and the advantages and disadvantages of using the technique when investigating modified RNA systems and how the experimental data generated may translate to a computational study are discussed. A summary of this section is outlined in Table 1.

### 4.1. Detection Methods

#### 4.1.1. Mass Spectrometry (MS)

MS can be used to chemically identify RNA modifications as well as sequence modified RNAs. MS requires a very little amount of sample (as low as attomolar concentrations), making the technique attractive to troublesome biological targets with low yields [96]. Yet, the sample must be pure as anything with a charge, such as salt ions and divalent metals, will be detected and increase the complexity of the spectra. 

There are three main methods one can use to analyze modified RNA by MS: top-down, bottom-up, and nucleoside MS [96]. Top-down analysis of RNA uses an RNA sample that has not been hydrolyzed and provides the total mass and identification of modifications including their location and sequence context [97]. An important assessment of top-down data is sequence coverage. Sequence coverage describes the number of cleavage sites where at least one resulting product could be detected. There are a fair number of techniques used for this method (CAD [98], EDD [99], RTD [100], AINETD [101], etc.) but the overall methodology remains the same: Fragment the structured ion and analyze the fragments to achieve overlapping coverage for the sequence. The major benefits of this method are the ability to perform de-novo sequencing, identify RNA modifications within the sequence, and the location of such modifications [96]. An advantage of this method is that most mass spectrometers are fully capable of performing this with acceptable accuracy and precision [96]. However, equal mass modifications (such as m1A, m6A, etc.) or “mass-silent” modifications (pseudouridine and uridine) cannot be immediately distinguished by mass and would require either additional separation methods or the ability to isolate and fragment the mass in question [96]. Data analysis and interpretation can be a hassle as it is not straightforward due to spectra complexity and lack of software. 

Bottom-up analysis typically uses partially hydrolyzed RNAs for mass mapping and to provide some sequence context, though this method does require a sequence to compare back to [102]. These partially hydrolyzed oligonucleotides can be separated and analyzed by tandem MS/MS. Oligonucleotides of length 5–15 nucleotides are desirable as this length will be unique yet small enough to decrease the complexity of data analysis. In recent years, there has been a push to identify and implement new RNases that can be used in combination to increase sequence coverage through the generation of overlapping digestion products [103,104,105]. However, as these RNases have been produced in-house in the labs that develop them, they suffer from a lack of reproducibility. 

Nucleoside MS of a fully hydrolyzed RNA can give chemical identities of modifications even at extremely low abundances [106,107]. Hydrolysis is typically achieved using endonucleases and phosphodiesterases and then the sample is subsequently dephosphorylated using alkaline phosphatase [106]. The resulting sample only contains the free nucleosides. These nucleosides can be separated by liquid chromatography, chemically identified by fragmentation methods, and quantified. However, the analysis of nucleoside digests has several disadvantages. First, the sample must be extremely pure, otherwise, the quantification of detectable RNA modifications will be affected [96]. Second, artifacts can be easily introduced due to the hydrolysis protocol [96]. Labile RNA modifications can be easily destroyed under the mild alkaline conditions [108]. Additionally, isocytidines may emerge through the amination/imination of carbonotiolated nucleosides [109]. Thirdly, the enzymes used to hydrolyze the RNA sample may not be capable of cleaving modified RNA [110,111]. 

MS experiments are sensitive to RNA modifications and can provide the chemical identity, sequence position, and the number of RNA modifications. Through -omics methodology, MS can provide these data via high throughput technologies for larger RNAs as well as heterogeneous biological samples (cell lysates, etc.) [112,113,114]. Additionally, advances have been made in native MS to provide tertiary contact information as well as the stability of certain folded RNAs [115,116,117,118]. Data from these experiments could be useful to computational studies, however, MS experiments take place within a vacuum. Gas phase force fields have not advanced enough to take advantage of this information when investigating modified RNA structure and dynamics. 

#### 4.1.2. Sequencing Techniques

RNA sequencing techniques are a rapidly developing field. The field is currently split into two generations: next generation and third generation. Both generations provide single nucleotide resolution, allowing the position of RNA modifications to be uncovered. However, these techniques may not provide the identification of the exact modification. 

Next generation sequencing (NGS) techniques rely upon various chemical treatments that affect particular RNA modifications, allowing them to be detected as either: a reverse transcription (RT)-stop (naturally or chemically induced), as a misincorporation of nucleotides into the cDNA, through chemically-induced cleavage of the backbone, or through antibody-based enrichment methods (MeRIP-Seq, i/miCLIP) [119,120]. Natural RT stops are visualized as an altered reading of the modification during primer extension. This can result in a full stop of the RT or misincorporations in addition to the aborted RT product. Chemically inducing RT stops is considered one of the more reliable methods of NGS [120]. By using certain chemical reagents, a treated sample can be compared to a mock (untreated) sample, and signals can be excluded or reduced, leading to the identification of a modified position [120]. Chemically-induced cleavage, or selective ligation, works very similarly and is dependent on the strength of the signal to indicate either enhanced cleavage or a protected site, both of which can indicate a modified position [120]. Antibody-based enrichment methods have been in use since the late 70s and are still in development today [119]. Cross-linking and immunoprecipitation (CLIP-Seq) has become popular to identify RNA-binding protein binding sites and has some functionality towards identifying the binding sites of RNA modifying proteins, such as writers and erasers [119,121,122,123]. After identifying the binding site of such proteins, the associated RNA can be sequenced and possible modification sequence positions uncovered [119,121,122,123]. However, this method is plagued by low affinity and specificity [120]. Additionally, enrichment methods for modified RNAs are lacking and there are multiple types of artifacts that can occur [96]. Still, this method is widely used for many modified RNA sequences. 

Third generation sequencing (TGS) takes advantage of single-molecule analysis. NGS involves amplification steps and provides only an average picture of the possible modified positions in a RNA sequence, whereas TGS techniques can provide the exact combination of modified sites for a given single RNA molecule. Two TGS techniques have been developed recently: PacBio SMRT technology [124] and Oxford nanopores [125]. PacBio SMRT technology uses zero-mode waveguide arrays to monitor single RNA molecules as they are sequenced [124]. Oxford nanopores carry out sequencing by predicting sequences from electric current patterns, which change as each nucleobase passes through the pore [125]. Both techniques suffer from the same pitfalls. Precision in both techniques is mediocre and data analysis can be arduous due to lack of appropriate data analysis software. 

Both NGS and TGS techniques can detect some RNA modifications and can provide a sequence position for RNA modifications within a modified RNA. TGS shows the most promise as both PacBio SMRT technology and Oxford nanopores analyze a single molecule and can passively detect a modification without interpreting an RT stop or misincorporation. NGS techniques are still under development regarding the detection of RNA modifications, however, there have been some recent successes combining CLIP with RNA-modifying enzymes to identify dihydrouridine positions across the transcriptome [126].

### 4.2. Structural Analysis Methods

#### 4.2.1. UV Optical Melting Experiments

Optical melting experiments using UV spectroscopy have been used for decades to determine thermodynamic data for RNA. The principle depends on a two-state model, where a double-stranded RNA is subjected to increasing temperatures that break the hydrogen bonds between base pairs, resulting in a single-stranded/unstructured RNA [127]. Relatively small amounts of RNA are needed (micromolar concentrations), the experiments are fast, and the instrumentation is inexpensive [127].

Optical melting experiments can provide melting temperature, enthalpy, entropy, and free energy changes for state changes including duplex formation [127]. These data indicate the stability of a helical structure and have become the core of nucleic acid secondary structure prediction algorithms [127,128]. In addition to optical melting experiments, other spectroscopic techniques such as Fourier Transform Infrared (FT-IR) spectroscopy [129,130,131], Raman spectroscopy [132,133,134,135,136], circular dichroism [137,138,139,140,141], and fluorescence-based techniques (microscale thermophoresis) [142,143,144,145,146,147,148] have also been employed to study RNA structure. Similar experimental information (melting temperature, helical stability, enthalpy, entropy, and free energy changes) can be gleaned from each and translated to computational endeavors. However, to keep this article concise, we will not go into further detail, but each technique has been reviewed elsewhere. RNA modifications have been known to affect base pairing, stacking, and the stability of the duplex structure, therefore, replicating this behavior in simulations would provide a more accurate modified RNA model. While there have been several studies on duplexes containing RNA modifications [149,150,151,152,153], they are by no means comprehensive over all naturally occurring RNA modifications. Additionally, optical melting experiments do not provide insight beyond helical stability, (e.g., tertiary junctions, ligands, protein binding, etc.), so insight into more complex modified RNA structures would be lacking.

#### 4.2.2. Nuclear Magnetic Resonance (NMR)

NMR is a powerful tool when investigating RNA structure and is particularly sensitive to the effects of RNA modifications on a modified RNA structure. The RNA of interest must be labeled (^13^C, ^15^N) to be detected, which requires some preparation and quite a bit of care must be taken to maintain the purity of both sample and structure to use NMR successfully [96]. NMR can detect protons, typically H, C, N, and P within RNAs. These proton signals can give several NMR data: NOE contacts, *J*-couplings, residual dipolar couplings, and cross-correlated relaxation rates [96]. Complete interpretation of these data will lead to a three-dimensional structure determination of an RNA sample. 

Because RNA modifications are chemically diverse compared to the canonical RNA nucleotides, their signals are easily recognized, typically occurring in regions of NMR spectra devoid of RNA canonical signals. In fact, early tRNA studies used the modified nucleotides as molecular probes to explore its 3D folding and stability [154,155]. In more recent years, NMR has been used for investigating the structural effects and changes in dynamics due to the presence of RNA modifications [156,157]. In this way, NMR can be considered more powerful than other structural techniques, such as X-ray crystallography, as it both captures structural and dynamics information. Recent novel approaches using NMR allow investigators to monitor tRNA maturation continually, and therefore, gain insight into tRNA modification events [158]. 

However, NMR does have a size limitation before the data becomes too complex to process. Solution state NMR studies have an intrinsic molecular weight limit of around 40 kDa or between 120 and 150 nt [159]. Solid state NMR experiments will be dependent upon the quality of the sample preparation, which has a direct effect on spectral linewidth and crowding [160]. Typically, anything larger than 50 nt will require nonuniform isotopic labeling strategies [160]. In addition, larger RNAs are often studied in sections, with the assumption that there are no long-range interactions between the sections [159]. 

NMR data can easily provide information about the secondary structure of RNA and identify base pairs and their types. However, if determination of the 3D structure is the goal, then a full assignment of RNA signals is necessary. This can be done through a hybrid approach using both bond experiments (HCP) and assignment of distance restraints (NOESY) [159]. NOEs can only be detected within 6Å and since RNAs tend to be long, flexible dynamic structures, it can be difficult to resolve the entire molecule [96]. The experimental NOEs can be used to generate a 3D model for use in further computational experiments. Yet, the generated 3D file from these coordinates can be biased due to the simulated annealing and subsequent refinement as the quality of the 3D file is completely dependent on the accuracy of the force fields used [96]. 

#### 4.2.3. X-ray Crystallography

X-ray crystallography is a well-known structural technique for biomolecules. However, the crystallization of RNA molecules is often more challenging than proteins. Analyzing modified RNA by X-ray crystallography is hampered by two major limiting factors: crystallization and phase problem [161]. RNA’s higher order folding landscape is often complex and contains kinetic traps, encouraging sample heterogeneity. To encourage better crystallization, RNAs of interest are typically altered to encourage crystal contacts, improve crystal packing, and discourage phase separation [161]. These alterations can include substituting the 2′ oxygen with selenium, adding “sticky ends” or hanging nucleotides as well as decreasing flexible areas of interest to only use a “minimal structure” [161]. Additionally, sequences might be altered to prevent crystal twinning. Phase problems interfere with the quality of phase information, which is critical to calculate the 3D structure of macromolecules after diffraction data is collected [161]. 

Experimentalists have developed a methodology to convert naturally occurring RNAs into sequences/structures that can crystallize [162,163,164]. This method focuses on a motif of interest, a hairpin for instance, and evaluates the surrounding sequence for highly variable regions. These regions are considered nonfunctional and therefore perfect for sequence alteration or subtraction to encourage crystallization [162,163,164]. However, RNAs for which the structures are solved via this approach are often generated using in vitro transcription and are inherently devoid of modified nucleotides. This altered composition can affect the results significantly, leading to a lack of desired insight into naturally occurring RNA structure and function. 

X-ray crystallography data can provide the three-dimensional coordinates of a biomolecule within a crystal. This is particularly helpful in determining the position and orientation of RNA modifications within a structure. However, as discussed above, very rarely are RNA modifications within a solved RNA crystal structure (which themselves are rare), and even so, the structure may not be considered ‘native’ due to the crystallization process. Therefore, the 3D coordinates provided by X-ray crystallography would not give insight into the modifications’ structural effect on the native, biologically relevant RNA. 

#### 4.2.4. Cryogenic Electron Microscopy (Cryo-EM)

Cryo-EM gathered attention as an alternative to X-ray crystallography and NMR for biomolecule structural determination as it removed the need for crystallization [165]. When a sample is analyzed by Cryo-EM, it is flash frozen and then irradiated with electron beams. The two-dimensional projection images are then recorded, typically providing an ensemble of many molecules in different orientations. In contrast to X-ray crystallography, Cryo-EM only requires microgram amounts of samples that are directly affixed on cryo grids after purifications, bypassing the need to form stable, homogeneous crystals [165,166]. In addition, Cryo-EM does not require detergents and solvents, which can destabilize or otherwise affect the structure of the biomolecule [165,166]. 

Cryo-EM data includes images collected on direct electron detectors that have several frames per image, increasing sensitivity and allowing for conformationally heterogeneous samples to be separated [167]. Cryo-EM still requires the cryo-grids to be well-populated with intact particles and due to the time between sample application to the grid and actual vitrification, preferential structures or aggregation can occur, creating an artifact within the data [167]. Troubleshooting these problems can take an extreme amount of time (months to years) for a single sample [167]. Additionally, data collection can take much longer than X-Ray crystallography due to data collection and lack of automation; data processing and analysis typically requires extensive computational time even when using parallel processing on GPUS [167]. 

However, the data provided by cryo-EM experiments directly translates to a 3D model. Additionally, if the signal to noise ratio is good and the refinement is of high quality, one can obtain several 3D models representative of different structural populations of RNA [168]. Since RNA modifications can influence RNA structure, this sensitivity can be key to discerning the subtleties of the folding pathway of modified RNA [168]. Additionally, multiple 3D models allow for a better understanding of the long-distance interactions within a larger modified RNA. Structural context and long-distance interactions are types of data that are unknown regarding RNA modifications, making cryo-EM data that much more valuable. 

### 4.3. Summary of Experimental Approaches for Modified RNA Research

**Figure 4:**
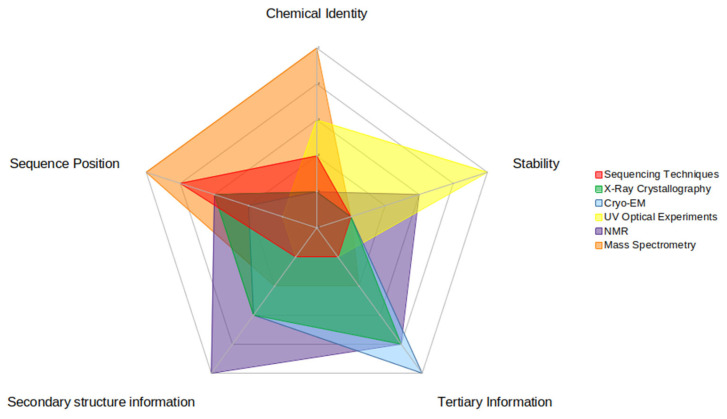
This figure illustrates the relative strengths and weaknesses of each experimental technique for each piece of data that is useful in computational investigations. There are five pieces of data highlighted here: (clockwise on the figure) chemical identity (of the RNA modification), stability (of the modified RNA structure), secondary structure information (of the modified RNA), tertiary information (of the modified RNA), and sequence position (of the RNA modification). Strengths are represented by higher numbers (towards the outside of the circle) while weaknesses are represented by lower numbers (inside of the circle). The relative strength score was based upon how much information the experimental technique could impart to each type.

Computational investigations into modified RNAs require experimental data to give context to the modifications’ effects on structure and dynamics. However, no one experimental technique can provide enough context to parameterize these modified nucleotides to effectively simulate a modified RNA (Figure 4). Therefore, the techniques described above are best used in concert to provide the most structural context. First, modifications must be reliably detected and identified within a sequence. MS and TGS techniques can provide primary sequence context for modified RNA, yet only MS can provide chemical identity. Then, the secondary structure needs to be determined, as h-bond-derived base-pairing drives the formation of RNA structure. NMR and UV optical melting experiments give insight into the secondary structure as well as the dynamics and stability of RNA motifs, such as hairpins and loops. Ultimately, 3D information is key to giving context to the effect of RNA modifications on structure. While X-ray crystallography has historically been a standard in structural techniques, RNA, in general, is more suited to other techniques such as Cryo-EM or NMR to provide tertiary context, where crystallization is not required. 

## 5. Perspective

In the past decade, interest in RNA modifications has skyrocketed due to improvements in detection and identification methods that have revealed them to play a much larger role in biology than previously assumed. However, a detailed understanding of their function remains elusive. In many cases, the presence and identity of specific modifications can be detected, but their actual effect on RNA folding and function is still poorly understood. Techniques such as X-ray crystallography, NMR, and Cryo-EM, which are all well-suited for obtaining high-resolution 3D structures of proteins, face challenges for characterizing RNA, whose structures are often unresolvable due to RNA’s inherent flexibility and dynamics. This creates a pressing need for accurate physics-based computer simulations of modified RNAs that could potentially provide atomistic insight into how modifications affect RNA structure and function. 

In this review, we highlighted some of the challenges faced by both experimental and computational approaches with a focus on how that affects our ability to model and simulate modified RNAs. In a more mature field, a review article such as this would be expected to give an overview of notable past successes as well as detailed established best practices in the field. However, when it comes to atomistic simulations of modified RNAs, the field is still very much in its infancy. At the current time, even unmodified RNAs of >20 nucleotides cannot be accurately folded de-novo using unbiased all-atom molecular dynamics simulations, and these are systems where incredibly abundant detailed biophysical information on their conformational thermodynamics is readily available. With so little that is experimentally known about how modifications affect RNA folding, dynamics, and molecular recognition, it is extremely difficult to ascertain if the behavior of a simulated modified RNA is realistic or not. This leads to an inherent chicken-and-egg problem (hence the graphical abstract), that on one hand simulations are needed to provide mechanistic insight into the behavior of modified nucleotides that are difficult to measure experimentally, and on the other hand, with absent detailed experimental measurements, we cannot meaningfully assess how accurate these simulations predictions are, much less calibrate them to improve their performance.

That said, MDS can still be utilized to provide useful insights in cases where the modifications occur in a well-defined structural context such as in a synthetic double-helix (i.e., nearest-neighbor thermodynamics [169]) or the long-known occurrences in tRNAs. However, for most modifications, answering even basic questions regarding modification induced conformation, stability, and interaction changes remains challenging. Unlike proteins, there have been very few success stories with regards to ab initio folding of RNA [62,170,171]. So, while one can obtain a set of parameters for modified RNA that are self-consistent with unmodified RNA parameters in a particular biomolecular force field (i.e., CHARMM or AMBER), this is necessary but not sufficient to prove they accurately depict the salient chemical properties of each modification. At best, the results can be considered “as good as” their canonical counterparts, which have not been able to fold anything more complex than tetraloop hairpins. This is not intended as a criticism, as it merely reflects the paucity of structural and thermodynamic data available for simulation developers to assess the accuracy of their models.

So where do we go from here? There are lessons that can be learned from the successes in protein simulations. The systematic improvement in protein force fields required widely agreed-upon test systems that were both computationally tractable and experimentally well-characterized, as detailed in Lindorff-Larson et al.’s review [172]. The frequent exchange of ideas between the simulation and experimental communities, which can result in clever, better ways to compare models vs measurements, is a second vital ingredient. Just as the Turner group used invaluable NMR experiments to gauge the accuracy of canonical RNA simulations [173,174], there is a need for similarly strategic experiments that could be used as a “Rosetta Stone” to both calibrate and assess the behavior of modified nucleotide simulations. 

## Figures and Tables

**Table 1:** Summary of techniques discussed: advantages, disadvantages, and the computational information for RNA modifications that can be gleaned from each.

Experimental Methods	Advantages	Disadvantages	Computational Information
Mass Spectrometry	Native solvent conditions Attomolar concentrations can be used	No 3D insight Sample is not recoverable Size limitations Gas phase experiments	Chemical ID Sequence position
Sequencing Techniques	Single nucleotide resolution Population or single molecule-based methods available	Mediocre accuracy and precision in detection	Sequence position
UV Optical Experiments	Micromolar concentrations can be used Fast experimentation Thermodynamics insight	Two state dependent No insight beyond helical stability	Melting temperature Helical stabilityChanges in free energy, enthalpy, and entropy
NMR	Native conditions Sensitive to structure fluctuations	Size limitation Lengthy data interpretation 3D molecule resolution difficult to attain	Distance restraints Nucleotide/RNA 3D orientation Secondary structure (base pairing/non-paired)
X-ray Crystallography	3D structure can be determined	RNAs are hard to crystallize Non-native conditions Requires homogeneous crystals	3D coordinates and orientation of RNA molecule
Cryo-EM	Heterogeneous populations detectable Crystals not necessary Native conditions	Data collection, analysis, and troubleshooting is lengthy and complex	3D coordinates and orientation of RNA molecule Tertiary contacts detectable

## Data Availability

Not applicable.
